# Alternating magnetic fields and antibiotics eradicate biofilm on metal in a synergistic fashion

**DOI:** 10.1038/s41522-021-00239-y

**Published:** 2021-08-12

**Authors:** Qi Wang, Jonathan Vachon, Bibin Prasad, Christine A. Pybus, Norman Lapin, Rajiv Chopra, David E. Greenberg

**Affiliations:** 1 Department of Radiology, UT Southwestern Medical Center, Dallas, TX USA; 2 Medical School, UT Southwestern Medical Center, Dallas, TX USA; 3 Department of Internal Medicine, Infectious Diseases and Geographic Medicine, University of Texas Southwestern Medical Center, Dallas, TX USA; 4 Advanced Imaging Research Center, UT Southwestern Medical Center, Dallas, TX USA; 5 Department of Microbiology, UT Southwestern Medical Center, Dallas, TX USA

**Keywords:** Health care, Biofilms

## Abstract

Hundreds of thousands of human implant procedures require surgical revision each year due to infection. Infections are difficult to treat with conventional antibiotics due to the formation of biofilm on the implant surface. We have developed a noninvasive method to eliminate biofilm on metal implants using heat generated by intermittent alternating magnetic fields (iAMF). Here, we demonstrate that heat and antibiotics are synergistic in biofilm elimination. For *Pseudomonas aeruginosa* biofilm, bacterial burden was reduced >3 log with iAMF and ciprofloxacin after 24 h compared with either treatment alone (*p* < 0.0001). This effect was not limited by pathogen or antibiotic as similar biofilm reductions were seen with iAMF and either linezolid or ceftriaxone in *Staphylococcus aureus*. iAMF and antibiotic efficacy was seen across various iAMF settings, including different iAMF target temperatures, dose durations, and dosing intervals. Initial mechanistic studies revealed membrane disruption as one factor important for AMF enhanced antibacterial activity in the biofilm setting. This study demonstrates the potential of utilizing a noninvasive approach to reduce biofilm off of metallic implants.

## Introduction

Metal implants such as prosthetic joints, bone fixation hardware, and dental implants, are widely used in medicine to replace damaged or diseased tissue^1^. In aggregate, millions of metal devices are implanted into humans every year globally^2^. In the case of total knee arthroplasty, over one million procedures are performed in the US each year, and the number is projected to reach ~3.5 million by the year 2030 due to population and health trends^3^. Approximately, 1–2% of these implants become infected. This serious complication is challenging to treat^3^. Currently, the treatment of prosthetic joint infections (PJI) mainly relies on multiple revision surgeries. Initial surgery is performed to remove the infected implant and a temporary spacer is placed^4^. Antibiotics are administered for several weeks to clear the residual infection. Once the patient is confirmed to be free of infection, a final surgery is performed to implant a new prosthesis^5^. Treatment of PJI is highly invasive with a significant negative impact on patients’ quality of life. Moreover, the failure rate of these multistage surgeries is currently over 10%^6,7^. In addition, the projected cost of treating PJI is 1.6 billion USD in 2020 in the United States alone, creating a significant economic burden to the health care system^8^.

A primary reason that antibiotic treatment of metal implant infections (MII) (such as PJI) is ineffective is due to the formation of biofilm on the implant surface^9^. Biofilm is a thin (tens to hundreds of micrometers) aggregate of bacteria and extracellular polymeric substances (EPS)^10^. EPS is generated by bacteria and forms a barrier to the surrounding environment, rendering these organisms up to 1000-fold more resistant to antibiotics as well as providing protection from the immune system^11^. Importantly, increasing antibiotic resistance only further complicates this problem. Aside from PJI, biofilm also plays an important role in the infection of other widely used medical implants, including catheters, mechanical heart valves, and bone fixation hardware^1,12,13^.

Nonsurgical means of eradicating biofilm would be a significant advance in the treatment of MII. Several physical approaches for eliminating biofilm have been proposed including electrical current^14–16^, ultrasound^17^, heat^18–20^, and shock waves^21^. However, these methods are either hard to apply in vivo or have limitations for use on metal implants. A potentially safer and more effective method of biofilm removal off of metal implants is through the use of alternating magnetic fields (AMF). AMF can be delivered from outside the body and does not suffer from penetration depth limitations or complex wave distortions through tissue boundaries. When metal implants are exposed to AMF, electrical currents are induced on the surface, resulting in the generation of heat. Previous studies have shown the feasibility and effectiveness of biofilm elimination by AMF^19,22^. After just a few minutes of AMF treatment, the biofilm on a stainless-steel washer was reduced significantly^22^.

However, the necessity to sustain temperatures ranging from 50 to 80 °C for several minutes to achieve biofilm reduction presents challenges for AMF to be utilized clinically. In addition, incomplete eradication of bacteria via AMF results in regrowth within a short period of time^22^. One approach to overcoming this obstacle is to consider combination therapy with antibiotics. In vitro studies have demonstrated a greater and sustained reduction in bacterial burden. As such, AMF and ciprofloxacin in combination were observed to be more effective than AMF or ciprofloxacin alone in reducing biofilm and prevented its recurrence for up to 24 h post treatment^20,23,24^. In addition, utilizing brief, intermittent AMF exposures could address the issue of elevated implant temperatures and safety. As shown previously in a murine model, elevating a metal implant to a target temperature quickly and for a brief period resulted in much less tissue injury compared to longer duration exposures^25^. Further, these short duration exposures can be delivered repeatedly with sufficient cool-down time in between exposures to allow for thermal doses that are therapeutic on the implant surface without a concomitant rise in tissue thermal dose. This approach is referred to as intermittent AMF (iAMF).

Here, we investigate the efficacy of iAMF exposures in combination with antibiotics to eliminate biofilm on metal surfaces in vitro. We determine the relationship between AMF parameters (temperature, duration, # of exposures) and antibiotics (drug, concentration, dosing). We explore this approach in both prototypic Gram-positive and Gram-negative pathogens and begin to explore the mechanisms that underlie this relationship by attempting to rescue multidrug-resistant pathogens (MDR) with iAMF.

## Results

### Characterization of the iAMF system

**Fig. 1:**
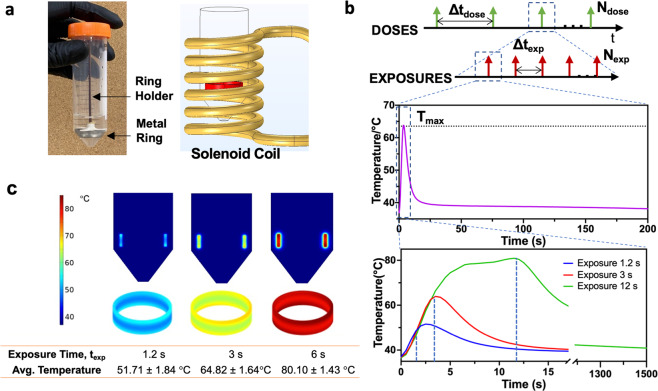
Simulation and measurements of intermittent alternating magnetic field (iAMF) heating. The experimental set-up consisted of a stainless-steel ring with biofilm in media in a 50-mL tube and held in place by a plastic holder (**a** left image). The tube is placed in a solenoid coil (**a** right image). b shows a representation of the iAMF dosing scheme. An iAMF treatment was organized as a series of doses separated by hours (Δ*t*_dose_, panel top). *N*_dose_ was the number of doses in the whole treatment. Each dose is composed of multiple short-term exposures of AMF (*N*_exp_). During the iAMF exposure, the AMF was on for several seconds of (*t*_exp_), and the rings were heated to the targeted temperature (*T*_max_). The exposures were delivered at intervals (Δ*t*_exp_) to allow the temperature to return to baseline (**b** middle image). Temperature vs. time for the ring upon AMF exposure to a *T*_max_ of 50, 65, and 80 °C is shown **c**. Simulated AMF heating of a metal ring for different exposure times depicts spatial temperature variation on the surface, and minimal heating of surrounding media. The mean and standard deviation of the temperature are shown.

iAMF exposures were produced using an in vitro system (Supplementary Fig. 1a, b) designed to heat metal rings with precisely controlled exposure durations, and with specified exposure and dosing intervals. The system is comprised of 32 identical solenoid coils, capable of generating a uniform AMF (10.2 ± 0.3 mT) at the center of each coil. In addition, the measured magnetic field agreed well with the predictions from simulation (11.2 ± 0.4 mT). Metal rings were chosen since they were heated uniformly in the magnetic field of a solenoid when oriented along the axis of the coil as shown in Fig. 1a. The finite-element simulation results in Fig. 1c depict the uniformity of heating that can be achieved. The surface temperature distribution on the rings after 1.2, 3, and 6 s of heating are shown, with uniform temperatures around the circumference of the ring, and a standard deviation of no more than 2 °C between the top and middle. Further, the simulations highlight that for this short duration of heating, the media surrounding the rings is not significantly heated, which was also observed by actual measurement (Supplementary Fig. 2b). Cumulative equivalent minutes at 43 °C (CEM43) are used for evaluating mammalian cell thermal damage^26^. Usually, 240 min is considered as the threshold for permanent damage in muscle tissue^27,28^. Because the heat transfer from the rings to the adjacent media is governed by heat conduction and convection, we calculated the CEM43 around the ring with the assumption that the ring was surrounded by muscle tissue (i.e., only heat conduction). The CEM43 did not exceed 240 min at 2 mm from the ring under iAMF with *T*_max_ = 80 °C and at 1 mm under 12 iAMF exposures of *T*_max_ = 65 °C, suggesting no permanent tissue damage at this distance (Supplementary Fig. 3).

### Elimination of biofilm by iAMF and antibiotics

**Fig. 2:**
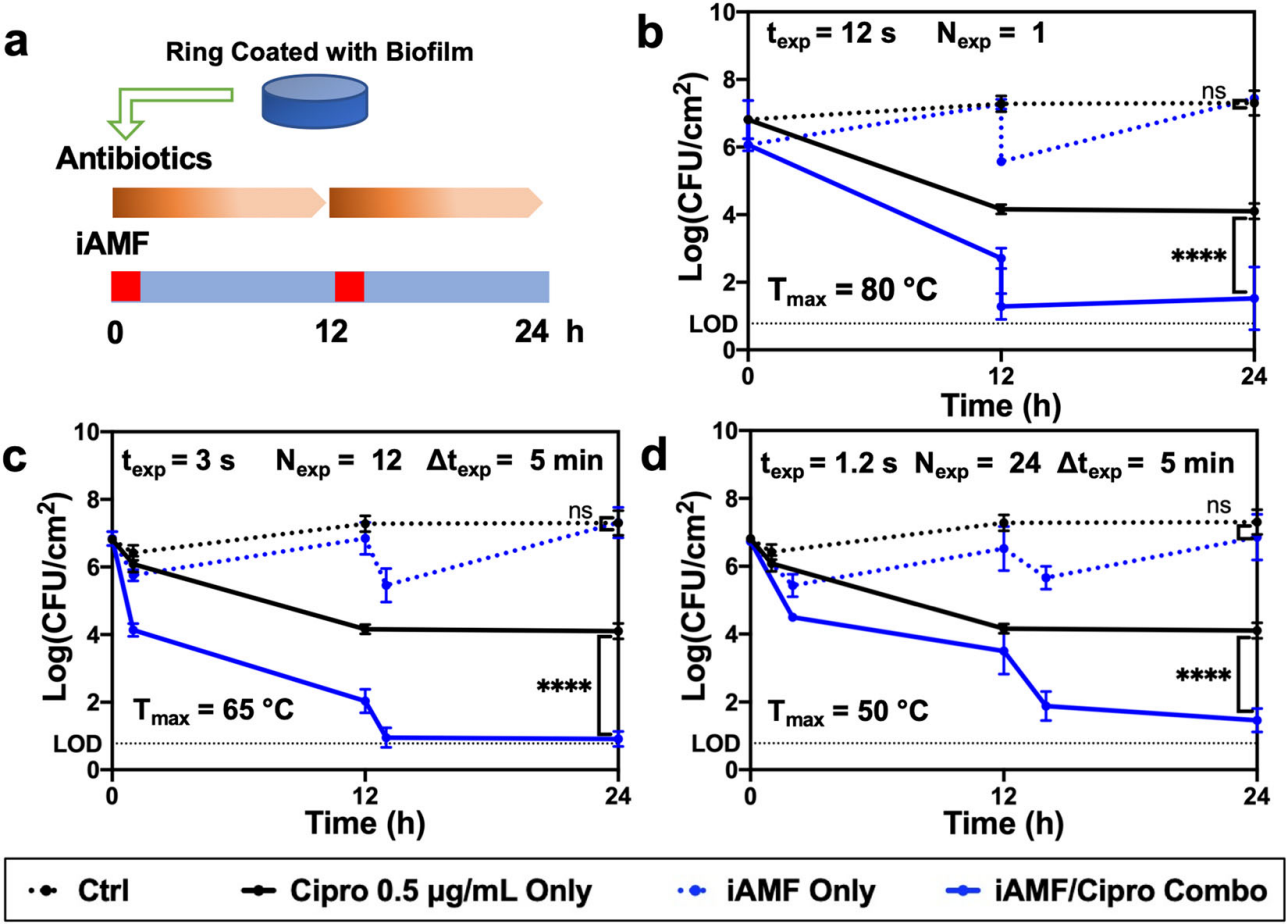
iAMF and ciprofloxacin are synergistic in reducing *P. aeruginosa* biofilm. **a** The general treatment scheme for combining iAMF and antibiotics. **b**–**d** Bacterial log reduction over a 24-h period for PAO1 biofilm upon treatment with iAMF heating alone (blue dotted line), ciprofloxacin at 0.5 μg mL^−1^ alone (black solid line) or iAMF + ciprofloxacin (blue solid line) at different peak temperatures *T*_max_ of **b** 80 °C, **c** 65 °C, or **d** 50 °C. The number of exposures was varied for each case as shown in the panels. Untreated controls (black dotted line) were not exposed to antibiotics or AMF. Colony-forming units (CFU) were counted at 0, 12 h (pre- and post-AMF), and 24 h post treatment. *n* = 3. Error bars indicate SD. CFU limit of detection (LOD) = 0.78 log(CFU cm^−2^). Two-way ANOVA. Significance: not significant (ns) and significance at *p* < 0.0001 (****).

Having characterized the dynamics of ring heating with the iAMF system, we investigated its ability to eradicate biofilm from the ring surface (Fig. 2). Each of the three iAMF treatments investigated (dotted blue lines) were able to reduce *Pseudomonas aeruginosa* PA01 biofilm by approximately 1–2 log after each dose. However, between doses, CFU levels reverted to baseline. The rings exposed to 0.5 μg mL^−1^ of ciprofloxacin alone (solid black line) showed a steady CFU reduction over the first 12 h of almost 3-log, followed by a plateauing after that. Strikingly, the iAMF exposures combined with ciprofloxacin (solid blue lines) demonstrated a consistent reduction in biofilm down to the limit of detection. The reduction in CFU immediately after each dose was equal or larger for combined therapy compared with iAMF alone. In between the AMF doses at time 0 and 12 h, there was a further reduction in CFU, presumably as ciprofloxacin demonstrated enhanced activity in biofilm. Of note, the CFU reduction at 0 and 12 h were of a similar magnitude suggesting a consistent AMF treatment effect after each dose. This trend was observed for three different treatment strategies in which the target temperature (*T*_max_), and a number of exposures (*N*_exp_) was altered. Furthermore, more exposures were required at lower temperatures to observe an equivalent reduction in biofilm after 2 doses (Fig. 2b, c, d). At 24 h, the difference in CFU between the combined treatment group and all other groups was highly significant (*p* < 0.0001). The same treatment strategy with iAMF at *T*_max_ = 65 °C and ciprofloxacin combined was conducted on equally sized plastic rings or Grade 5 titanium rings with *P. aeruginosa* biofilm. On plastic rings, biofilm CFU showed no significant difference when treated with iAMF and ciprofloxacin compared to ciprofloxacin incubation alone (Supplementary Fig. 4). For biofilms on titanium rings, a material that is widely used in medical implants, biofilm reduction from iAMF and ciprofloxacin treatment was similar as that seen on stainless steel rings (Supplementary Fig. 5).

To evaluate whether a synergistic relationship exists between heat and antibiotics on biofilm, an experiment was conducted using a temperature-controlled water bath. Biofilms were exposed to varying durations of heating at specified temperatures, and then the CFU reduction in bacteria in the presence and absence of various antibiotic concentrations was quantified (see Supplementary Methods). The MBEC (minimal biofilm eradication concentration) was used to quantitatively study the synergistic effect of heat and ciprofloxacin as previously described^29^. The results demonstrated synergy with fractional inhibitory concentration (FIC) index values that were below 0.5 (the definition for synergy) for various combinations of heat treatment time and ciprofloxacin concentrations at both 12 and 24 h post single heat treatment^30,31^. This suggests that heat and ciprofloxacin display synergistic activity in the biofilm setting (Supplementary Fig. 6)^29^.

**Fig. 3:**
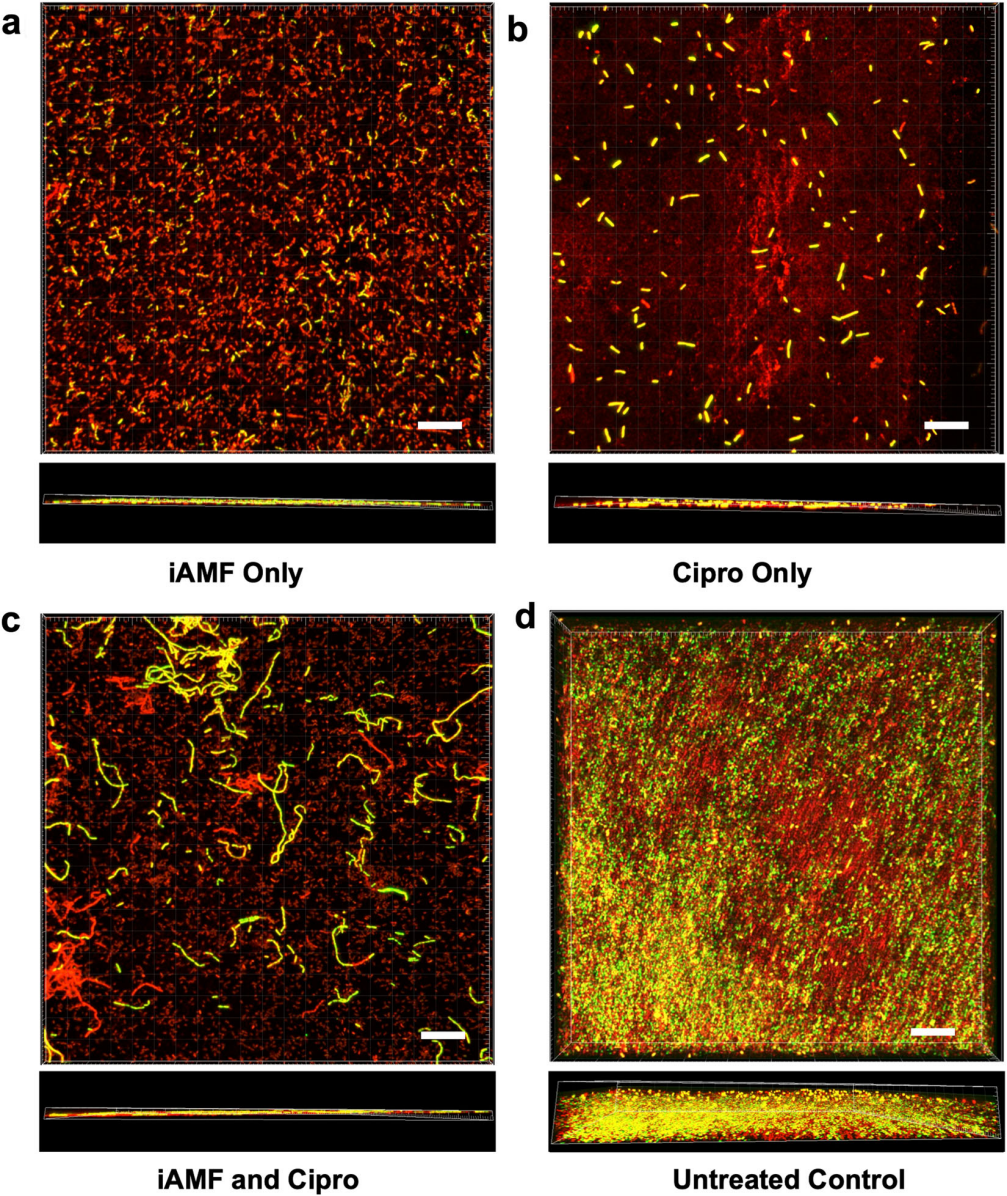
iAMF and ciprofloxacin cause bacterial morphologic changes. Laser scanning confocal microscopy of *P. aeruginos*a (PAO1) biofilm-infected rings 12 h post start of treatment (top and side views). Bacteria within the biofilm express a green fluorescent protein (GFP) while EPS are stained with ConcanavalinA-Alexa Fluor 647 conjugate, fluorescing red. Rings were **a** treated with iAMF (*T*_max_ = 65 °C) for 1 h, then incubated in MHII media for 12 h, **b** incubated in ciprofloxacin at 0.5 μg mL^−1^ for 12 h or **c** treated with 1 h iAMF while incubating with 0.5 μg mL^−1^ of ciprofloxacin for 12 h. **d** Untreated control. Scale Bar: 100 μm.

The enhanced reduction in biofilm to combined iAMF and antibiotics was also observed visually utilizing laser scanning confocal microscopy (Fig. 3). GFP-PAO1 biofilms were treated using iAMF (*T*_max_ = 65 °C, Δ*t*_exp_ = 5 min, *N*_exp_ = 12) and 0.5 μg mL^−1^ ciprofloxacin. GFP-PAO1 cells are represented in green and ConcanavalinA-Alexa Fluor 647 stained EPS was shown as red. This allowed for the morphology of bacterial cells to be observed under different treatment conditions. With ciprofloxacin only (Fig. 3b), the bacteria showed slight elongation compared to iAMF only (Fig. 3b) and control (Fig. 3d) at 12 h post-treatment. While the iAMF only group displayed diffuse ConcanavalinA-Alexa Fluor 647 stained EPS, the combined treatment of iAMF and ciprofloxacin (Fig. 3c) had less dense EPS staining. In addition, there were increased numbers of GFP-expressing cells that were elongated, a visual representation of *Pseudomonas* during quinolone treatment^32,33^.

**Fig. 4:**
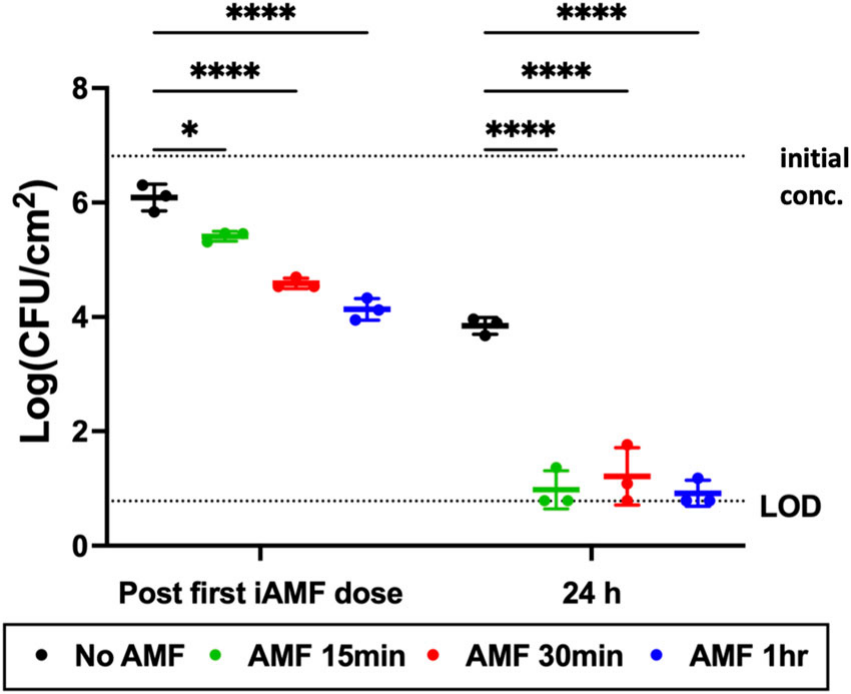
iAMF displays dose-dependent reductions of *P. aeruginosa* biofilm in combination with ciprofloxacin. iAMF doses (*T*_max_ = 65 °C, Δ*t*_exp_ = 5 min) were delivered at 0 and 12 h with 3, 6, or 12 exposures in each dose (incubated with 0.5 μg mL^−1^ of ciprofloxacin). Colony-forming units (CFU) were counted immediately after the first iAMF dose (left) and at 24 h post treatment (right). For treatment with ciprofloxacin alone (post first dose for no AMF) CFU was counted after 1 h in ciprofloxacin. The CFU at time 0 was 6.81 log(CFU cm^−2^). *n* = 3. Error bars indicate SD. CFU limit of detection (LOD) = 0.78 log(CFU cm^−2^). Two-way ANOVA. Statistical significance: *p* = 0.0318 (*) and *p* < 0.0001 (****).

The impact of iAMF dose duration was investigated in more detail. *P. aeruginosa* biofilms were treated with iAMF (*T*_max_ = 65 °C) for dosing durations that ranged from 15 min to 1 h in combination with 0.5 μg mL^−1^ ciprofloxacin following the same treatment scheme as in Fig. 2a. Exposures were spaced apart by 5 minutes in each of the treatments. Immediately after combined iAMF and antibiotic treatment, reduction in CFU demonstrated a dose-dependent response with longer durations of iAMF resulting in greater decreases (Fig. 4, *p* = 0.0318 for 15 min iAMF and *p* < 0.0001 for 30 and 60 min iAMF). After 15 min of iAMF there was a 1.41 log reduction that increased to a 2.68 log reduction after the 1 h dose. After 24 h, there was a 2.7 log reduction in biofilm treated with ciprofloxacin only, whereas the combination therapy achieved a greater than 5 log reduction, approaching the limit of detection for all iAMF treatment durations (*p* < 0.0001 for all the three dosing durations). These results showed that biofilm can be effectively eliminated through combined treatment of iAMF and ciprofloxacin at a variety of dosing durations. Indeed, only three 3-s iAMF exposures over 15 min together with ciprofloxacin were sufficient to effectively eliminate *P. aeruginosa* biofilm.

**Fig. 5:**
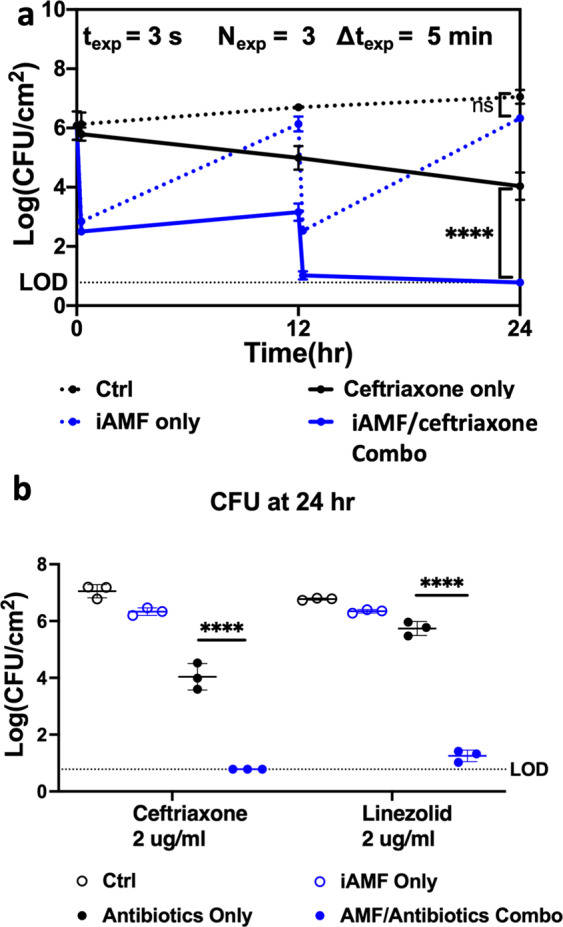
iAMF and antibiotics are synergistic in reducing *S. aureus* biofilm. *S. aureus* (UAMS1) biofilm was treated with iAMF doses at 0 and 12 h (*T*_max_ = 65 °C, Δ*t*_exp_ = 5 min, 15 min per dose) and specified antibiotic. **a** Biofilm log reduction (CFU) post 24 h with iAMF and 2 μg mL^−1^ ceftriaxone. CFU was counted at time points 0, 12 (pre- and post-iAMF), and 24 h. **b** CFU of *S. aureus* biofilm 24 h post-treatment with iAMF and ceftriaxone (2 μg mL^−1^) or linezolid (2 μg mL^−1^). *n* = 3. Error bars indicate SD. CFU limit of detection (LOD) = 0.78 log(CFU cm^−2^). Two-way ANOVA. Statistical significance: not significant (ns), and significance at *p* < 0.0001 (****).

Similar patterns were observed for iAMF and antibiotic treatment of *Staphylococcus aureus* biofilm. In addition to being a Gram-positive pathogen with several structural and metabolic differences compared to *P. aeruginosa*, *S. aureus* has clinical importance as one of the more common pathogens associated with MII. *S. aureus* (UAMS1) biofilms were treated with iAMF and antibiotics alone and in combination. Two antibiotics commonly used clinically were selected: ceftriaxone (2 μg mL^−1^) and linezolid (2 μg mL^−1^). These concentrations represented the minimum inhibitory concentration (MIC) for this strain. As in previous experiments, iAMF doses were delivered at 0 and 12 h. Each dose was composed of iAMF exposures with the following specifications: *T*_max_ = 65 °C, Δ*t*_exp_ = 5 min, *t*_dose_ = 15 min. For treatment with iAMF and 2 μg mL^−1^ ceftriaxone (Fig. 5a), biofilm CFU initially decreased by over 3 logs, suggesting that *S. aureus* biofilm has a greater sensitivity to iAMF dosing alone (3.29 log reduction) compared with *P. aeruginosa* (0.96 log reduction) with the same 15 min iAMF dose. As observed with PA01, in between doses, biofilm CFU returned to control levels for iAMF only groups. Incubation with ceftriaxone alone only led to approximately a 2-log reduction after 24 h. However, CFU reduction was significantly larger after 24 h when treated in combination with iAMF (*p* < 0.0001) with CFU approaching the limit of detection. At 24 h, iAMF and ceftriaxone (2 μg mL^−1^) or iAMF and linezolid (2 μg mL^−1^) showed significantly lower CFU than with antibiotics alone (Fig. 5b; *p* < 0.0001 ceftriaxone and for linezolid).

**Fig. 6:**
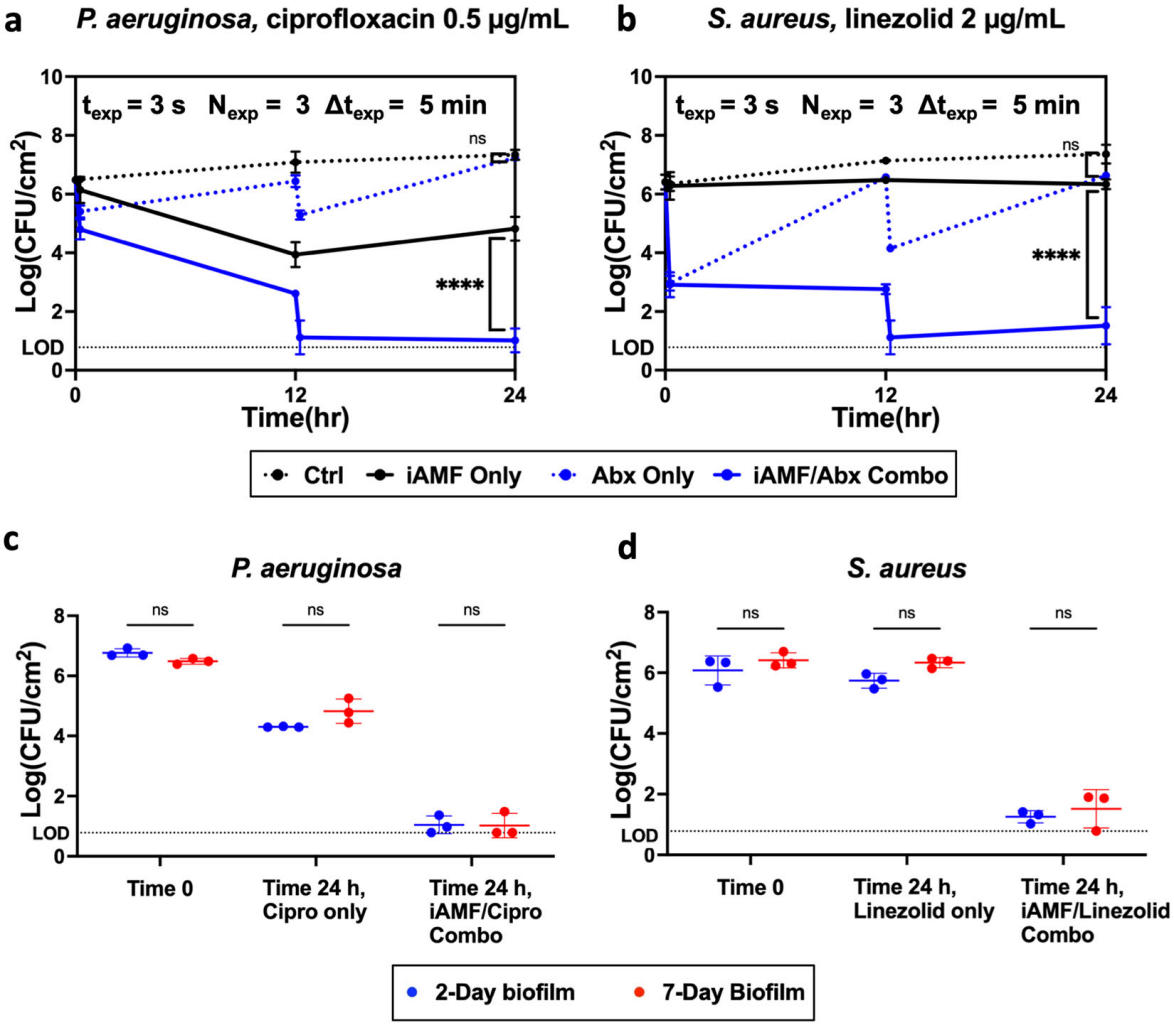
iAMF and antibiotics can work on biofilm of various ages. *P. aeruginosa* (PAO1) and *S. aureus* (UAMS1) biofilms were cultured until day 7 following the same protocol with media replenishment every 24 h. Then the biofilms were treated with iAMF doses at 0 and 12 h (*T*_max_ = 65 °C, Δ*t*_exp_ = 5 min, 15 min per dose) and specified antibiotics. **a** 7-day *P. aeruginosa* (PAO1) biofilm log reduction (CFU) post 24 h with iAMF and 0.5 μg mL^−1^ ciprofloxacin. CFU was counted at time points 0, 12 (pre- and post-AMF), and 24 h. **b** 7-day *S. aureus* (UAMS1) biofilm log reduction (CFU) post 24 h with iAMF and 2 μg mL^−1^ linezolid. CFU was counted at time points 0, 12, and 24 h. **c** Comparison of prior 2-day (48 h) biofilm and 7-day *P. aeruginosa* (PAO1) biofilm under the same iAMF (*T*_max_ = 65 °C, Δ*t*_exp_ = 5 min, 15 min per dose) treatment and 0.5 μg mL^−1^ ciprofloxacin at time 0 and 24 h. **d** Comparison of 2-day (48 h) biofilm and 7-day *S. aureus* (UAMS1) biofilm under the same iAMF (*T*_max_ = 65 °C, Δ*t*_exp_ = 5 min, 15 min per dose) treatment and 2 μg mL^−1^ linezolid at time 0 and 24 h. *n* = 3. Error bars indicate SD. CFU limit of detection (LOD) = 0.78 log(CFU cm^−2^). Two-way ANOVA. Statistical significance: not significant (ns).

The age of the biofilm can vary in real-life clinical situations. We investigated if the combination of iAMF and antibiotics could eliminate more mature biofilms beyond 48-h (2-day) old ones. 7-day *P. aeruginosa* (PAO1) and *S. aureus* (UAMS1) biofilms were cultured and the same experimental conditions were performed with iAMF at *T*_max_ = 65 °C as for 2-day biofilms. Similar reductions in CFU to 2-day biofilms were seen. When treated with the same iAMF dose (*T*_max_ = 65 °C, Δ*t*_exp_ = 5 min, *t*_dose_ = 15 min) as used with the 2-day biofilm and antibiotics (0.5 μg mL^−1^ ciprofloxacin for PAO1, and 2 μg mL^−1^ linezolid for UAMS1), the CFU change followed the same trend as was seen previously (Fig. 6a, b). There was no significant difference in the magnitude of the reduction of biofilm to iAMF and antibiotics for 2 and 7-day biofilms (Fig. 6c, d).

### Specific resistance mechanisms determine synergy between iAMF and antibiotics

**Fig. 7:**
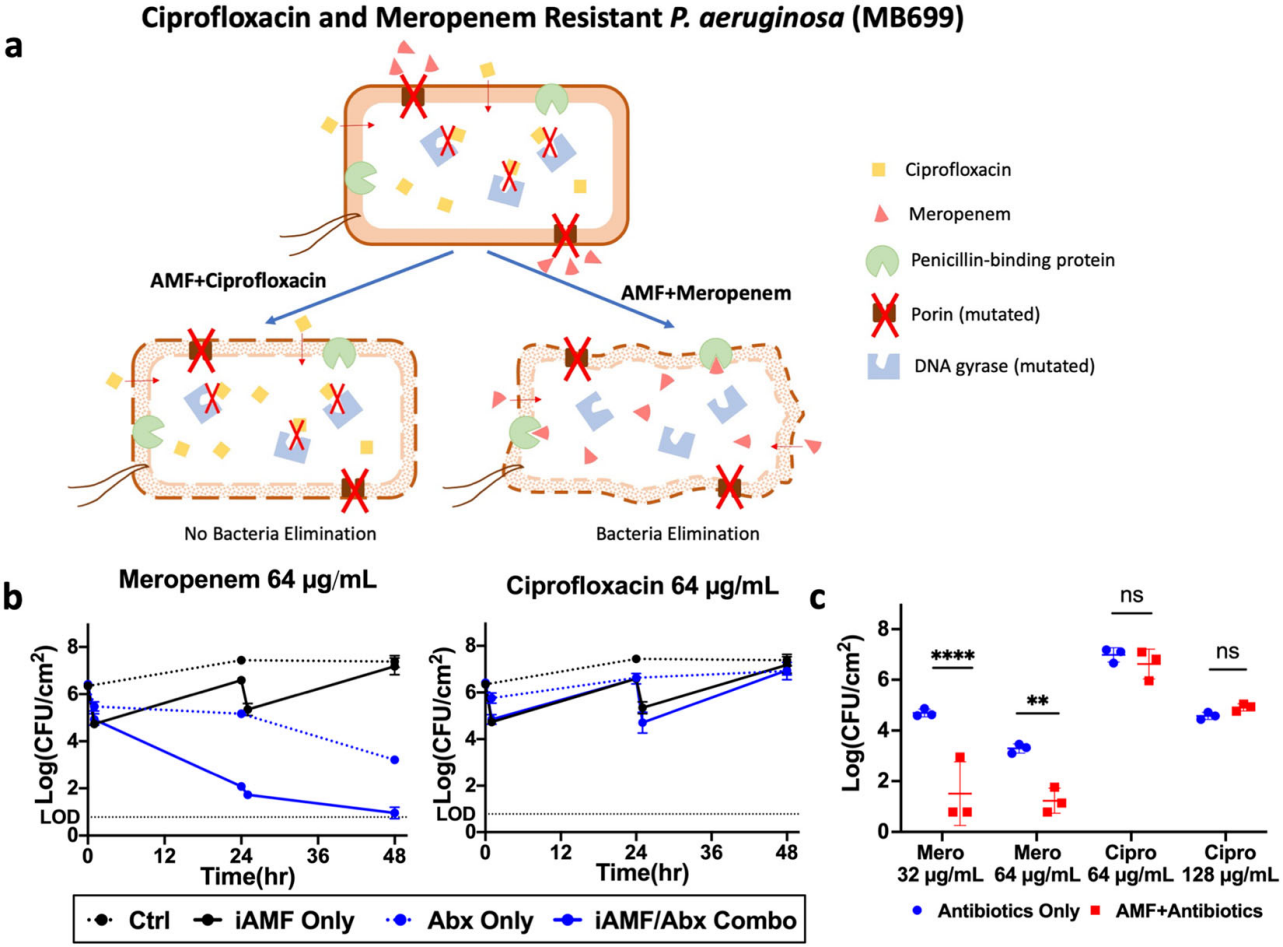
iAMF can rescue MDR pathogens depending on the mechanism of resistance. MDR *P. aeruginosa* (MB699) biofilm was treated with meropenem (MIC 64 μg mL^−1^) or ciprofloxacin (MIC 64 μg mL^−1^) with or without iAMF (dosed at 0 and 24 h, *N*_exp_ = 12, *T*_max_ = 65 °C, Δ*t*_exp_ = 5 min) while incubating with antibiotic for 48 h. **a** Proposed mechanism for sensitization of antibiotic-resistant biofilm to meropenem by AMF. **b** Treatment time course with meropenem (left) or ciprofloxacin (right) at 64 μg mL^−1^. Colony-forming units (CFU) were counted at time points of 0, 24, and 48 h. **c** Log reduction of antibiotic-resistant biofilm at different concentrations of ciprofloxacin or meropenem at 48 h post start of treatment. *n* = 3. Error bars indicate SD. CFU limit of detection (LOD) = 0.78 log(CFU cm^−2^). Two-way ANOVA. Statistical significance: not significant (ns), *p* = 0.001 (**), and *p* < 0.0001 (****).

Antibiotic resistance is becoming increasingly common. MDR only further complicates the treatment of biofilm-associated implant infections. The mechanism of the synergistic response between antibiotics and iAMF remains unknown. We hypothesized that one possible mechanism could relate to heat-induced membrane disruption allowing for increased uptake of the antibiotic. To test whether iAMF could enhance antibiotic activity in MDR pathogens and rescue activity of specific antibiotics depending on the resistance mechanism present, we utilized an MDR *P. aeruginosa* isolate (MB699) that was genomically and phenotypically characterized. This clinical isolate was genome sequenced as described previously^34^. It is an MDR isolate with a MIC of 64 μg mL^−1^ for both ciprofloxacin and meropenem. Analysis of the genome revealed mutations in DNA gyrase (*gyrA*, p.Thr83Ile) and topoisomerase IV (*parC*, p.Ser87Leu), which are associated with ciprofloxacin resistance, as well as loss of function mutations in the porin *oprD*, that are associated with carbapenem resistance. It was hypothesized that iAMF would enhance the activity of meropenem but not ciprofloxacin. MB699 biofilm was treated with iAMF using the following parameters: *T*_max_ = 65 °C, Δ*t*_exp_ = 5 min, *N*_exp_ = 12, *N*_dose_ = 2, Δ*t*_dose_ = 24 h. Antibiotic administration followed the same protocol as for the PAO1 experiments and each antibiotic was dosed at its MIC (Supplementary Table 3). After two doses (0 and 24 h) and determining CFU at 48 h, bacterial burden approached the limit of detection for treatment with iAMF and meropenem, while ciprofloxacin and iAMF did not result in a further reduction of CFU compared to either iAMF or antibiotic alone (Fig. 7a). The rescue of meropenem with iAMF was also seen at sub-MIC concentrations (32 μg mL^−1^) as well (*p* < 0.0001; Fig. 7b). Increasing the concentrations of ciprofloxacin did not lead to enhanced CFU decreases in combination with iAMF. The effects of iAMF and meropenem versus ciprofloxacin on MB699 were observed by scanning electron microscopy (SEM). At 12 h post-treatment of MB699 biofilm with iAMF (*T*_max_ = 65 °C, Δ*t*_exp_ = 5 min, *N*_exp_ = 12) and continuous incubation with 64 μg mL^−1^ of ciprofloxacin or meropenem, biofilms were fixed as described and imaged. For treatment with ciprofloxacin, meropenem, or iAMF alone, no obvious morphological changes were observed in the bacteria. With iAMF and ciprofloxacin, some changes were observed, with slight lengthening of bacteria and increased wrinkling of the membrane. Treatment with iAMF and meropenem displayed fragmented and deformed bacterial cells (Supplementary Fig. S7).

## Discussion

Although the effects of heat on bacterial killing have been known for years, major hurdles exist in order to utilize heat for antibacterial effects in the human body. Studies conducted by our group and others have demonstrated a strong therapeutic effect of heat generated via AMF and antibiotics on the eradication of biofilm^20,23,24^. A previous study by our group demonstrated that *P. aeruginosa* biofilm was more susceptible to ciprofloxacin after AMF treatment^22^. Pijls et al.^24,35^ reported similar results as was seen in this study, that there was an enhanced effect with AMF and antibiotics in *Staphylococcus epidermidis* and *S. aureus* biofilms on titanium alloy than with either treatment alone. One concern for the clinical adoption of AMF relates to therapeutic index, specifically the ability to reduce biofilm through thermal effects while minimizing neighboring tissue damage. In this study, we developed a method, intermittent AMF, that could deliver AMF to infected metal implants that could aid in moving towards these goals of maintaining efficacy while limiting any toxicity. The premise of iAMF is that brief exposures to the surface of an implant with sufficient cool-down time in between exposures will result in a therapeutic dose capable of eradicating biofilm while protecting surrounding tissues from damage.

We demonstrate that even iAMF exposures of a few seconds can reduce biofilm burden by 1–2 log in vitro. However, in the absence of more frequent dosing, there is regrowth back to baseline within 12 h. While more frequent dosing with iAMF could be used, an alternative approach would be to use iAMF to enhance the activity of antibiotics. As has been previously reported, the antibiotics used in this study were not affected by the heat generated by iAMF and maintain stability at these temperatures^36,37^. In combination, iAMF and antibiotics resulted in a dramatic decrease in biofilm burden over either treatment alone. Importantly, this effect was not limited to one pathogen or one antibiotic. We demonstrated that both clinically important Gram-positive (*S. aureus*) and Gram-negative pathogens (*P. aeruginosa*) and various antibiotics had their activity enhanced with iAMF. As diseases such as PJI are caused by a number of different bacterial pathogens, one goal of developing iAMF is to have a treatment that is efficacious regardless of the pathogen that is found. We also demonstrated that the combination of iAMF and antibiotics can effectively eliminate biofilms of different ages. Importantly, this treatment effect was not seen on plastic rings, indicating the underlying principle of current generation between AMF and metals. In addition to the quantitative reduction in bacterial burden, microscopy qualitatively supported the enhanced impact that iAMF and antibiotics had.

Biofilms are recalcitrant to antibiotic therapy for a number of reasons. This includes the difficulty in getting adequate concentrations of the drug to the target (bacteria) embedded within the biofilm matrix as well as difficulty in immune cells reaching these pathogens. This creates an environment where a biofilm-associated pathogen can be functionally antibiotic-resistant. The increasing rate of antibiotic resistance that is being seen worldwide will only further complicate the treatment of biofilm-associated infections. One of the most striking findings of this study was the ability to rescue certain multidrug-resistant bacteria based on the mechanism of resistance. We utilized a genomically and phenotypically characterized *Pseudomonas* strain to begin to understand what the mechanism of action is that explains iAMF synergy with antibiotics. One hypothesis is that iAMF disrupts bacterial membranes. If this is indeed the case, then it might be possible to rescue an MDR strain with an antibiotic if the mechanism of resistance was membrane-based (i.e., porins or efflux mechanisms). However, chromosomally based mechanisms of resistance (i.e., gyrase mutations) would not be impacted by an iAMF and antibiotic combination compared to either one alone. Our studies supported this hypothesis. We were able to show a synergistic effect with iAMF and meropenem in this MDR strain with known mutations in the porin *oprD* but not with ciprofloxacin as the strain contained DNA gyrase *gyrA* and topoisomerase IV *parC* mutations. Although, there are other potential mechanisms that could explain the interactions between iAMF and antibiotics in the biofilm setting, this data supports that membrane disruption is likely one important component. Future studies will more deeply investigate the mechanisms of antibiotic and AMF interactions.

Although we effectively eliminated biofilm using iAMF with antibiotics on metal implants in vitro, there are still some limitations of this study. Because of the treatment design of iAMF, determining the synergy of iAMF and antibiotics in the biofilm context is challenging. However, our water bath experiments combined with defining heating exposure time as the “dose” of an antimicrobial did in fact support that synergistic interactions between iAMF and antibiotics are being seen. Also, as this study was only performed in vitro, it is not clear how iAMF and antibiotics will translate in vivo. These studies are currently ongoing. Finally, it remains unclear whether the non-heat-related component of iAMF, specifically current deposition itself, has any role in disrupting biofilm.

There remain a number of unknowns regarding the ultimate deployment of iAMF in the clinical setting. This includes the optimal number of doses of iAMF that would lead to a durable treatment response as well as the optimal target temperature that would maintain efficacy while minimizing any potential safety concerns. Future and ongoing studies include exploring iAMF for safety and efficacy in a large animal model of implant infection. The translation to real-life medical implants could be challenging: first, the positioning of the implant may vary for different patients, even between treatments for the same patient, which can lead to inconsistent treatments. Second, because of the complexity of the implant, it can be difficult to achieve uniform heating for predictable biofilm elimination, which requires a more sophisticated coil design customized for a particular implant. In addition, other possible mechanisms of this interaction remain to be explored. This includes determining whether heat activates or results in stress response in pathogens in a way that makes them more responsive to particular antibiotics. Finally, current studies are focused on determining the response of iAMF and particular antibiotics in various strains of the same genus and species in order to determine the range of CFU reduction that will likely be achieved under various parameters. The hope is in the not too distant future, a non-invasive approach such as iAMF could be used with antibiotics to treat PJI without removal of the infected implant.

## Methods

### In vitro AMF system

A custom-designed system composed of multiple solenoid coils was constructed to deliver programmed AMF exposures to stainless-steel rings with existing biofilm held in 50 mL conical tubes. The parameters of AMF exposure were assigned using custom-developed software operating on a personal computer. A function generator (33250A, Agilent Technologies) was used to produce an RF signal. The signal was input into a 1000 W RF amplifier (1140LA, Electronics & Innovation), and the amplified signal was directed to the appropriate coil using a USB-controlled relay system. Each coil was constructed using 0.25-in. diameter copper tubing formed into a 6-turn solenoid with 1 cm pitch between turns (Fig. 1a). The coil diameter was chosen to accommodate a 50 mL conical tube holding the infected ring and media. A plastic holder was included in each conical tube to hold the ring in place, so the orientation was maintained across all coils. The coils were driven electrically as a parallel resonant circuit using a capacitor selected to tune the resonant frequency to approximately 500 kHz. The working frequencies of the coils ranged from 507 to 522 kHz. A matching inductor was also included in series with the resonant circuit to transform the impedance of each coil to 50 ohms for efficient power transfer. The complete system included four insulated boxes each containing eight coils, enabling the treatment of up to 32 samples with iAMF in a single experiment (Supplementary Fig. 1). The coils worked at 8 Vpp with a 50% duty cycle (100 ms per 200 ms) for the experiments described in this paper. A circulating fan with an integrated heater (Miller Manufacturing, MN, USA) was also incorporated into each box to keep the samples at 37 °C during extended-length experiments.

The strength of the AMF in the coil was characterized using a commercial 2D magnetic field probe (AMF Lifesystems, Inc., MI, USA). A Rogowski current probe (TRCP3000 current probes, Tektronix Inc., OR, USA) was used to measure the electrical current through the coils during operation.

To characterize AMF heating, uninfected metal rings were exposed for varying durations to reach desired maximum temperatures. The temperature of each ring exposed to AMF was measured using a fiber-optic temperature sensor (PRB-G40-2M-STM-MRI, Osensa Innovations, Burnaby, BC, Canada) attached to the center of the inner surface of the ring with high-temperature epoxy (Epotek 353ND, Epoxy Technologies, CA, USA. Tests were performed to confirm that the epoxy was unaffected by the AMF and did not produce false heating. See Supplementary Methods. Supplementary Fig. 2a). Ring temperatures were recorded at a rate of 2 Hz using a laptop computer. The temperature change during iAMF of media was also measured by placing the thermal sensor located in the center of the ring immersed in media (Supplementary Fig. 2b). The use of fiberoptic temperature sensors enabled accurate temperature characterization during AMF exposures since they are immune to electromagnetic interference.

Finite element simulations were performed using the commercial simulation software COMSOL Multiphysics (Comsol v5.5, Los Angeles, CA, USA) to model the interaction between AMF and a metal implant and to study the uniformity and magnitude of AMF-induced heating. A quasi-static approximation of Maxwell’s equation and Penne’s bioheat transfer model was used for electromagnetic and thermal simulations. The thermal dose is calculated as cumulative equivalent minutes (CEM43)^38^which gives the time–temperature relation in equivalent minutes as

1 $${\rm{CEM43}}=\mathop{\int}\limits_{t_0}^{^t{\rm{fina}l}}{R^{43-T(t)}dt}$$

where, *R* is the temperature dependence of the rate of cell death (*R* = 0.5 for *T* > *43*, *R* = 0.25 for 43 ≥ *T* ≥ 39), *dt* is the time interval, *t*_o_ and *t*_final_ are initial and final heating periods respectively in minutes. The thermal toxicity due to implant heating is determined based on the tissue damage radius CEM 240 min (irreversible damage)^27,28^ from the implant surface.

Figure 1a shows the 3D physical model used for simulation of the metal ring in aqueous biological media in the coil. The coil geometry and current measured in the section above were used for 3D modeling and initial conditions of 37 °C were selected for simulations. The physical properties used for simulations are listed in Supplementary Table 1^25,39^. Simulations were performed using free tetrahedral meshing with boundary layers. Grid independent studies were performed from coarser to finer meshes, settling on an optimal number of 186,634 elements to be used for analysis.

### In vitro AMF treatment

iAMF treatment parameters for the treatment were determined. The structure and timing of iAMF treatments are shown in Fig. 1b. Treatments were organized as a series of doses each separated by a fixed time (Δ*t*_dose_). The length of an iAMF dose ranges from 15 min to a few hours. *N*_dose_ is the number of doses in the whole treatment. Each iAMF dose is composed of multiple AMF exposures. During each exposure, AMF is on for a few seconds and the rings are heated. The exposures are separated by fixed time intervals (Δ*t*_exp_) to allow rings to cool to the initial temperature between exposures. (*N*_exp_) is the number of exposures performed in one iAMF dose. The heating from a typical exposure is shown with a specified target temperature, *T*_max_, and a cool down back to the baseline temperature over 3–5 min. The temperature profile for three different *T*_max_ values (50, 65, and 80) are also shown. The target temperatures were achieved by varying the duration of AMF exposure in the coil. For iAMF treatments at *T*_max_ = 80 °C, the temperature reached 80 °C in 6 s and was held until 12 s during the initial construction of the system. Therefore, this iAMF heating pattern was used in the *T*_max_ = 80 °C iAMF experiments described below.

Biofilm was grown on stainless steel rings (316 L, 3/4″ OD, 0.035″ wall thickness, 0.2″ height, cut from McMaster Carr, P/N 89785K857, USA) or Titanium rings (Grade 5, 3/4″ OD, 0.035″ wall thickness, 0.2″ height, cut from McMaster Carr, P/N 89835K93, USA) using the Gram-negative pathogen *P. aeruginosa* (PAO1: ATCC strain. PAO1-GFP: provided by Joanna Goldberg, MB699: provided by Sam Shelburne) or Gram-positive pathogen *S. aureus* (UAMS1, provided by M. Smeltzer). For *P. aeruginosa* biofilm, an isolated colony was inoculated into 3 mL of cation-adjusted Mueller Hinton II (MHII) media (Becton-Dickinson by Thermo-Fisher Scientific) and incubated at 37 °C for 18 h at 220 RPM. A working solution was made by adding culture to sterile phosphate-buffered saline (PBS). The bacterial concentration was adjusted with MHII using a UV spectrophotometer (Genesys 20, Thermal Scientific) at 600 nm until the optical density (OD) read between 0.07 and 0.08, indicating a concentration of ~ 10^8^ CFU mL^−1^. The working solution was then diluted to obtain a bacterial concentration of 5 × 10^5^ CFU mL^−1^. Biofilm was prepared on each metal ring by placing the ring in 5 mL of the bacterial solution in a 50 mL conical tube. The submerged ring was then incubated at 37 °C for 48 h at 110 RPM in a shaking incubator (Innova42, New Brunswick Scientific). Media was replenished midway at 24 h by exchanging the solution with 5 mL of fresh MHII. Biofilm prepared with *S. aureus* followed the same protocol using Tryptic Soy Broth (TSB, Becton-Dickinson by Thermo-Fisher Scientific). Biofilms other than the 7-day old biofilm in this study were prepared using this protocol. For the 7-day old biofilm, the rings were cultured similarly but the culture time was prolonged to 7 days with media replenishment every 24 h.

The biofilms were prepared, treated, and quantified as follows. The multi-coil system described above was used to investigate the response of biofilm (*P. aeruginosa* or *S. aureus*) grown on stainless-steel rings to AMF. Biofilm-coated rings were transferred to 50 mL conical tubes each with 10 mL fresh media containing antibiotics at set concentrations. Prior to the transfer, the tubes of fresh media were pre-warmed in the multi-coil system to 37 °C. After the rings were transferred to the tubes, sterile 3D-printed ring holders were placed on the top of the rings to maintain their orientation in the coil during AMF exposures. The rings were then exposed to intermittent AMF according to treatment protocols. After each intermittent dose, the rings were rinsed in 10 mL fresh antibiotic-containing media to remove planktonic bacteria. Then the rings were transferred again to 10 mL of fresh antibiotic-containing media and incubated at 37 °C. After a fixed time period (typically 12–24 h), the rings were exposed to a second dose of AMF using the same protocol, and the rings were again incubated in 10 mL media with antibiotics at 37 °C for another 12–24 h. Before and after each iAMF dose, and at the treatment endpoint, the rings were harvested and rinsed in 5 mL PBS and then transferred to 4 mL PBS. The rings were sonicated in an ultrasonic water bath for 5 min and bacterial density on the ring surface was quantified by plating on blood agar plates (TSA w/sheep blood, Thermo Fisher Scientific) using a standard serial dilution drip method. Three biological replicates were obtained for each experimental condition, and three technical replicates were utilized per experiment. Control groups for all studies included rings unexposed to antibiotics or AMF, and rings exposed to iAMF or antibiotics as monotherapy. All control groups went through the multiple rinse and transfer steps to account for any bacterial loss. A two-way ANOVA model was used to compare bacterial burden at different time points for single or combined therapy.

A final control group involved iAMF treatment of infected plastic rings with the same dimensions as the metal rings, to establish the observed effects were arising from the interactions between AMF and metal. See Supplementary Information for further details.

Experiments were performed with different AMF target temperatures (*T*_max_). Three unique iAMF treatment algorithms were delivered to rings infected with PA01 biofilm. The rings were incubated with ciprofloxacin (0.5 μg mL^−1^) in 10 mL MHII media at 37 °C for all treatments. Each treatment reached a different target temperature and had a different number of exposures in each dose, as described in Supplementary Table 2. Doses were repeated at 0 and 12 h.

Although multiple parameters were varied in each setting, the goal was to balance the maximum temperature with the number of exposures to maintain a level of safety. Each of these AMF treatment combinations was predicted to be safe in terms of tissue damage around the implant base on simulation (Supplementary Fig. 3).

Experiments with variable AMF dose durations in combination with antibiotic treatment were also conducted. Biofilms of *P. aeruginosa* strain PAO1 were prepared on stainless steel rings using the same culturing protocol as above and incubated with 0.5 μg mL^−1^ of ciprofloxacin in 10 mL MHII media at 37 °C. Rings were exposed to iAMF to a *T*_max_ of 65 °C with an exposure interval of 5 min. The duration of each iAMF dose ranged from 15 min to 1 h (3–12 exposures). Doses were delivered at 0 and 12 h and ring biofilm burden was quantified at various time points as above. For *S. aureus* experiments, a biofilm of UAMS1 was prepared on stainless steel rings according to the culturing protocol and incubated with 2 μg mL^−1^ of ceftriaxone or 2 μg mL^−1^ of linezolid, in 10 mL TSB media. The rings were exposed to iAMF to a *T*_max_ of 65 °C with 5 min between each exposure, for a duration of 15 min per dose (3 exposures). Doses were delivered at 0 and 12 h and biofilm burden were quantified at 24 h.

Biofilms of MB699, an MDR-strain of *P. aeruginosa*, were incubated with ciprofloxacin (64 or 128 μg mL^−1^) or meropenem (32 or 64 μg mL^−1^) in 10 mL MHII media. The rings were exposed to iAMF to a *T*_max_ of 65 °C with 5 min between exposures for a duration of 1 h per dose. Doses were delivered at 0 and 24 h and ring biofilm burden was quantified at 48 h.

### Imaging

Laser scanning confocal microscopy was performed on biofilms during iAMF treatment. Biofilms cultured from green-fluorescent protein (GFP) expressing PAO1 *P. aeruginosa* (GFP-PAO1) were prepared on rings using the above protocol, then exposed to iAMF (*T*_max_ = 65 °C, Δ*t*_exp_ = 5 min, dosing duration 1 h) and incubated in 10 mL MHII media with 0.5 μg mL^−1^ ciprofloxacin for 12 h. After rinsing in 5 mL DPBS, rings were then fixed in 5% glutaraldehyde (Sigma Aldrich, St. Louis, MO) at 37 °C for 30 min and protected from light. Rings were then rinsed in 5 ml of DPBS to remove excess glutaraldehyde and incubated in 200 μg mL^−1^ ConcanavalinA-Alexa Fluor 647 conjugate (Life Technologies, Grand Island, NY) for 15 min at room temperature in the dark to stain the EPS. After staining, rings were mounted on a 50 mm glass-bottom plate, and images were captured with a Zeiss LSM880 Airyscan laser confocal microscope. The GFP-PAO1 bacteria and ConcanavalinA-Alexa Fluor 647-stained EPS were imaged using a 40× objective lens. Multiple regions of the ring surface were randomly selected, and Z-stacks were acquired with slice step size of 0.5 μm. Before image processing, the z-stacks were deconvolved using Autoquant ×3 (Media Cybernetics, MD, USA) to improve the image resolution in *X*, *Y*, and *Z* directions. The deconvolved images were analyzed with Imaris x64 9.1.2 (Bitplane AG, Zurich, Switzerland).

SEM was conducted as follows. Biofilms cultured from *P. aeruginosa* (MB699) were prepared on rings and exposed to iAMF (*T*_max_ = 65 °C, Δ*t*_exp_ = 5 min, dosing duration 1 h) and incubated in 10 mL MHII media with 64 μg mL^−1^ ciprofloxacin or 64 μg mL^−1^ meropenem for 12 h. Then the rings with biofilm were prepared for SEM, following a similar protocol described previously^40^. The rings were carefully transferred to 4 mL PBS, rinsed in 4 mL of 0.1 M sodium cacodylate buffer three times, and fixed for 24 h in 4 mL of 2% glutaraldehyde, 2% paraformaldehyde in 0.1 M sodium cacodylate buffer. After rinsing in 4 mL of cacodylate buffer three times, the samples were re-fixed in 4 mL of 2% osmium in 0.1 M sodium cacodylate buffer for 2 h. Then the rings were further rinsed with 4 mL of deionized water five times and dehydrated at room temperature in five steps by placing the rings in 4 mL of 50%, 70% (twice), 85%, 95% (twice), and 100% ethanol, respectively for 5 min per solution. The rings were then transferred to 4 mL of 25%, 50%, 75%, and 100% (twice) hexamethyldisilazane (HMDS) in ethanol consecutively for 15 min each. Finally, the samples were left to dry for 24 h in a fume hood. The specimens were mounted on aluminum stubs, gold/palladium sputter-coated, and examined using a Zeiss Sigma VP scanning electron microscope. The images were acquired at 10 kV with a magnification of approximately 35,000×.

### Statistics

Significance was determined as described for in vitro AMF treatment by two-way ANOVA followed by Tukey’s multiple comparisons test. The “*n*” indicates the number of biological replicates. 2 or 3 technical replicates were conducted for each biological replicate. All analyses were performed using GraphPad Prism version 8.4.3 (San Diego, CA), and a *p*-value of < 0.05 was considered statistically significant.

### Reporting summary

Further information on research design is available in the Nature Research Reporting Summary linked to this article.

## Supplementary information

Supplementary Information

Reporting Summary

## Data Availability

The datasets generated during the current study are available from the corresponding author on reasonable request.
