# Supplementary material for: Alternating magnetic fields and antibiotics eradicate biofilm on metal in a synergistic fashion
Source: NPJ Biofilms Microbiomes. 2021 Aug 12;7:68. doi: 10.1038/s41522-021-00239-y (PMC8360946; doi:10.1038/s41522-021-00239-y)
Supplement: Supplementary file 1 — Supplementary Information [file 41522_2021_239_MOESM1_ESM.pdf]

Alternating Magnetic Fields and Antibiotics Eradicate Biofilm on Metal in a Synergistic Fashion

Supplementary Information

Qi Wang[1], Jonathan Vachon[2], Bibin Prasad[1], Christine Pybus[3], Norman Lapin[1], Rajiv Chopra[1,4], David E. Greenberg[3, 5] *

Affiliations:

[1]Department of Radiology, UT Southwestern Medical Center; Dallas, Texas 75390, USA.

[2]Medical School, UT Southwestern Medical Center; Dallas, Texas 75390, USA.

[3]Department of Internal Medicine, Infectious Diseases and Geographic Medicine, University of Texas Southwestern Medical Center; Dallas, Texas 75390, USA.

[4]Advanced Imaging Research Center, UT Southwestern Medical Center; Dallas, Texas 75390, USA.

[5]Department of Microbiology, UT Southwestern Medical Center; Dallas, Texas 75390, USA.

*Corresponding author. Email: david.greenberg@utsouthwestern.edu

Methods

Determining epoxy immunity (Epotek 353ND) to iAMF

A fiberoptic thermal sensor was glued with the Epotek 353ND epoxy at the tip and placed in 10 mL of DPBS. A bare sensor was placed in the DPBS as well. The distance between the tips of the two sensors was 1 cm. iAMF (T_{max} = 65 °C) was applied for 10 min and the temperature reading from the two sensors was recorded and compared.

Determination of synergy between heat and antibiotics in biofilm

The synergy of heat and ciprofloxacin in biofilm was determined using the fractional inhibitory concentration (FIC) index[1–3]. The FIC index was calculated based on the minimal biofilm eradication concentration (MBEC), defined as the lowest concentration of an antimicrobial substance that eradicates 99.9% of biofilm-embedded bacteria (3-log reduction in CFU mL^{-1}) compared to growth controls. The thermal treatment time was treated as the antimicrobial substance dose, and the MBEC for heat treatment was defined as the shortest treatment time that eliminated 99.9% of biofilm-embedded bacteria[4]. Thus, the equation for the FIC index calculation with heat treatment and antibiotics can be derived: $FIC = (C_{Heat}/MBEC_{Heat}) + (C_{Abx}/MBEC_{Abx})$, where $MBEC_{Heat}$ and $MBEC_{Abx}$ are the MBECs of heat treatment and antibiotics concentration alone, respectively, and C_{Heat} and C_{Abx} are thermal treatment time and antibiotics concentration in combination, respectively. FIC values of ≤ 0.5 were considered to be a synergistic effect, values of > 0.5 and < 4 indicated no interaction or additivity, and values of ≥4 indicated an antagonistic effect [3,4].

A temperature-controlled water bath (Model 1235, VWR Scientific) was used to conduct the heat treatment. 50 mL tubes with 10 mL fresh MHII were placed in the water bath and pre-

warmed to 65 °C containing ciprofloxacin at certain concentrations. PAO1 biofilms were prepared as described before. PAO1 biofilm-coated rings were transferred to pre-warmed 50 mL conical tubes and exposed in heated media for the targeted duration of time. After the heat exposure, the rings with biofilm were immediately transferred to 10 mL fresh media with ciprofloxacin in 50 mL conical tubes at set concentrations at 37 °C. Then the rings were incubated at 37 °C. After 12 h or 24 h, the rings were harvested and rinsed in 5 mL sterile PBS and then transferred to 4 mL PBS. After sonicating for 5 min in an ultrasonic bath, the bacterial density on the ring was enumerated using standard serial plating methods to determine the CFU cm^{-2}.

References

1. den Hollander, J. G., Mouton, J. W. & Verbrugh, H. A. Use of Pharmacodynamic Parameters To Predict Efficacy of Combination Therapy by Using Fractional Inhibitory Concentration Kinetics. *Antimicrob. Agents Chemother.* **42**, 744–748 (1998).
2. Berenbaum, M. C. A Method for Testing for Synergy with Any Number of Agents. *J. Infect. Dis.* **137**, 122–130 (1978).
3. Habash, M. B., Park, A. J., Vis, E. C., Harris, R. J. & Khursigara, C. M. Synergy of Silver nanoparticles and aztreonam against pseudomonas aeruginosa PAO1 Biofilms. *Antimicrob. Agents Chemother.* **58**, 5818–5830 (2014).
4. Dall, G. F. *et al.* Unexpected synergistic and antagonistic antibiotic activity against Staphylococcus biofilms. *J. Antimicrob. Chemother.* **73**, 1830–1840 (2018).

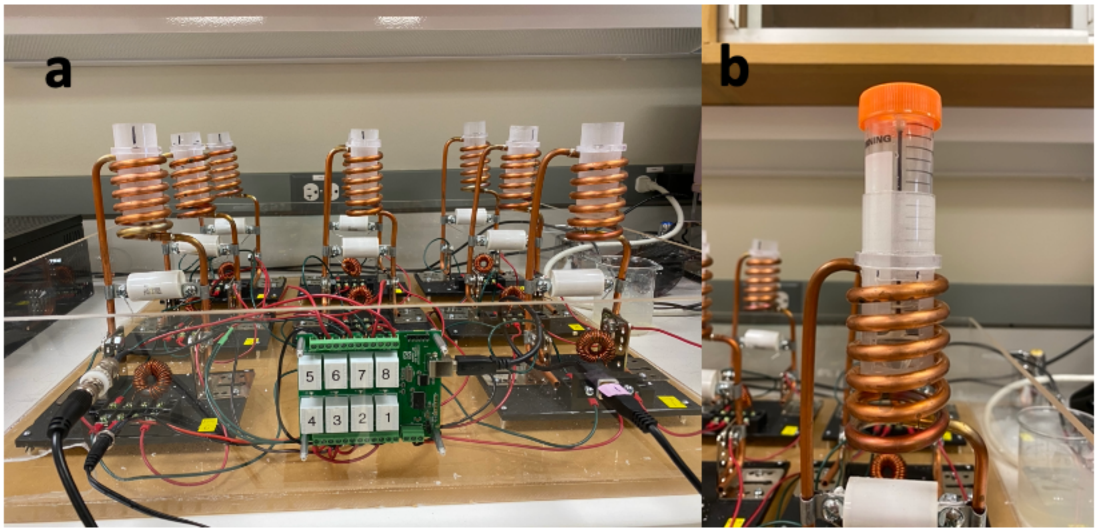

Supplementary Figure 1 Photo of the iAMF system. a eight coils which can be independently controlled to deliver iAMF. Four identical boxes were built, and 32 samples can be treated at the same time. A Styrofoam box (not shown) with temperature control was placed on top of the multicoil to maintain the temperature at 37 °C. **b** a conical tube with ring was place in the coil for treatment. The height of the tube was calibrated to position the ring in the center of the coil.

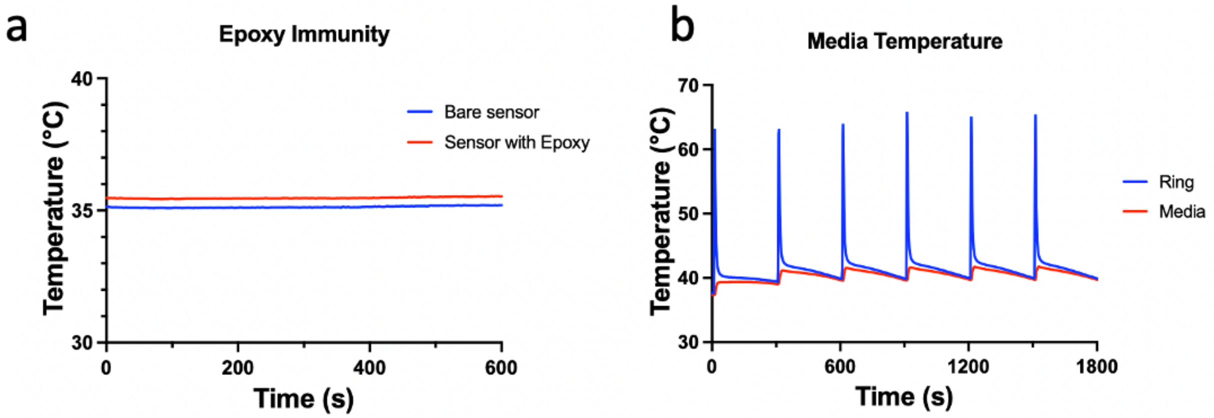

Supplementary Figure 2 Temperature change of epoxy and media under iAMF. a A fiberoptic thermal sensor was glued with Epotek 353ND at its tip and placed in 10 mL of DPBS

in a 50 mL conical tube, parallel with a bare sensor. The two sensors were treated with iAMF (T_{max} = 65 °C, Δt_{exp} = 5 min) to test if the Epotek 353ND was affected by iAMF. **b** A fiberoptic thermal sensor was placed in the center of the ring to evaluate the temperature change of media during iAMF (T_{max} = 65 °C, Δt_{exp} = 5 min) treatment.

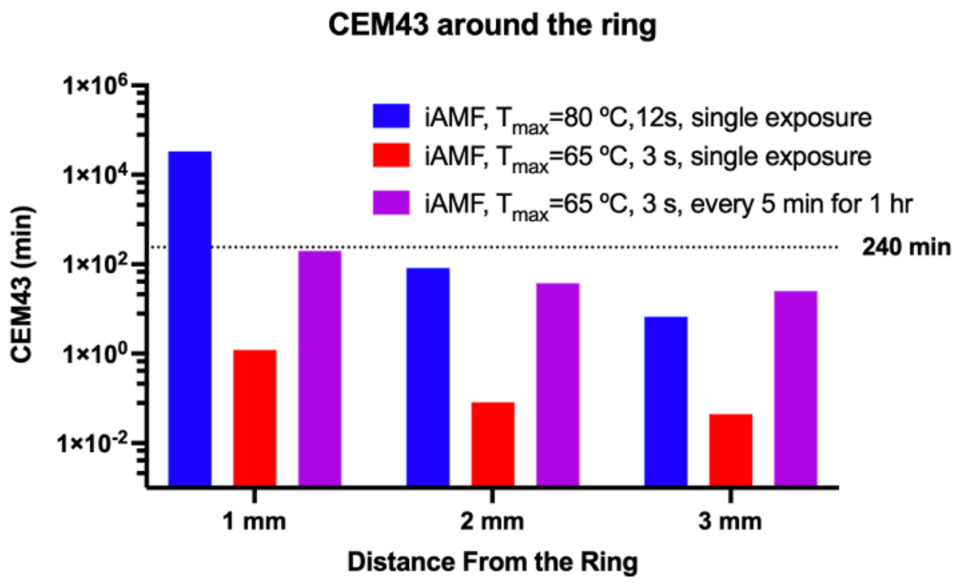

Supplementary Figure 3 CEM43 measurements surrounding the ring during iAMF. With the assumption that the rings were surrounded by muscle tissue, a simulation was performed to calculate the CEM43 at different distances from the ring with iAMF. Three iAMF treatment conditions were used: N_{exp} = 1, T_{max} = 80 °C; N_{exp} = 1, T_{max} = 65 °C; N_{exp} = 12, T_{max} = 65 °C, Δt_{exp} = 5 min. 240 min indicated the threshold of non-reversible cellular damage.

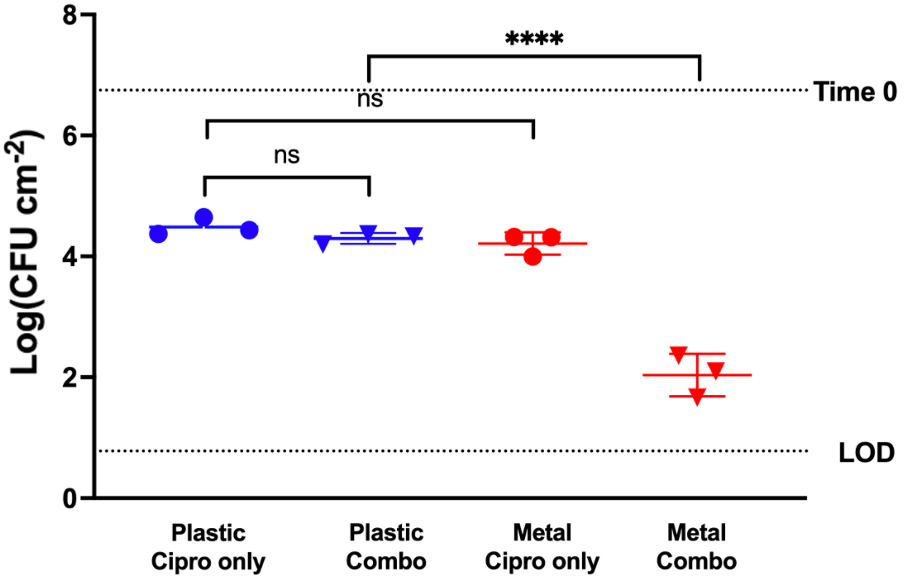

Supplementary Figure 4 Combined iAMF/antibiotic treatment of *P. aeruginosa* (PAO1) biofilm grown on plastic and stainless steel rings with 0.5 µg mL^{-1}ciprofloxacin. Biofilm was treated with iAMF doses (dosing duration 1 h, T_{max} =65 °C, Δt_{exp} = 5 min) and 0.5 µg mL^{-}1 ciprofloxacin at 0 h. Colony forming units (CFU) were counted at 12 h. Error bars indicate SD. CFU limit of detection (LOD) = 0.78 log(CFU/cm^2). One-way ANOVA. Statistical significance: not significant (ns) and significance at $p < 0.0001$ (****).

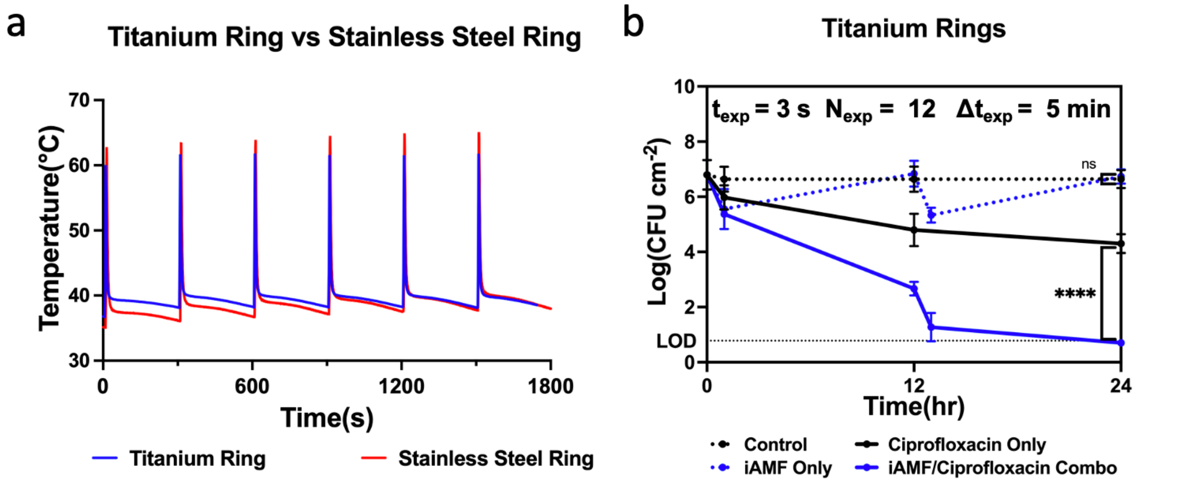

Supplementary Figure 5 Titanium rings compared to stainless steel rings during iAMF treatment. a The temperature change of titanium rings and stainless steel rings with iAMF (T_{max} = 65 °C, Δt_{exp} = 5 min) treatment. **b** *P. aeruginosa* (PAO1) biofilms were cultured following the same protocol. Then the biofilms were treated with iAMF doses at 0 and 12 h (T_{max} =65 °C, Δt_{exp} = 5 min, 15 min per dose) and 0.5 µg mL^{-1} ciprofloxacin. CFU were counted at time points 0, 12 (pre- and post-AMF) and 24 h. n=3. Error bars indicate SD. CFU limit of detection (LOD) = 0.78 log(CFU cm^{-2}). Two-way ANOVA. Statistical significance: not significant (ns), and $p < 0.0001$ (****).

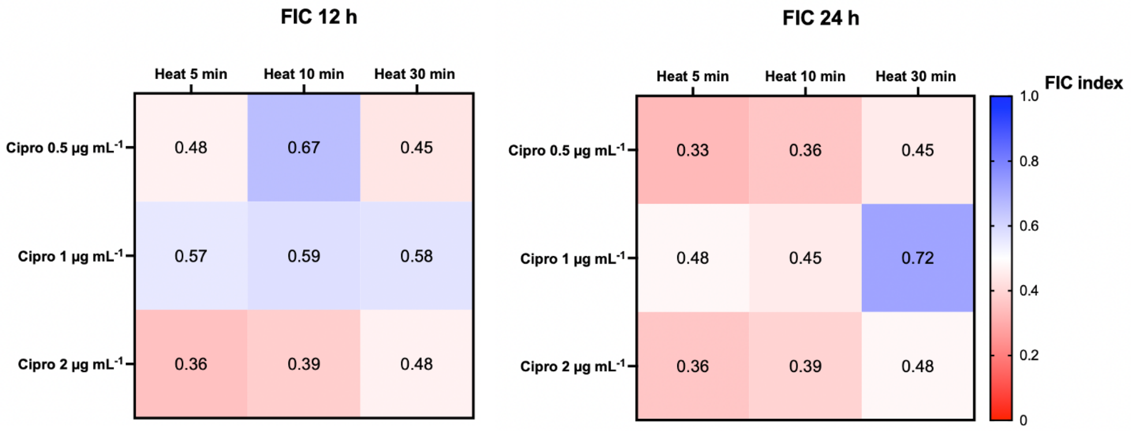

Supplementary Figure 6 FIC index of thermal treatment time and ciprofloxacin concentrations for biofilms. PAO1 biofilms were treated at 65 °C at time 0 for certain time periods, and incubated with ciprofloxacin at various concentrations for 12 h or 24 h at 37 °C. The numbers in the heat map showed the FIC index values for the treatment combination. FIC values of ≤ 0.5 were considered to be a synergistic effect, values of > 0.5 and < 4 indicated no interaction or additivity, and values of ≥4 indicated antagonistic effect. n=3.

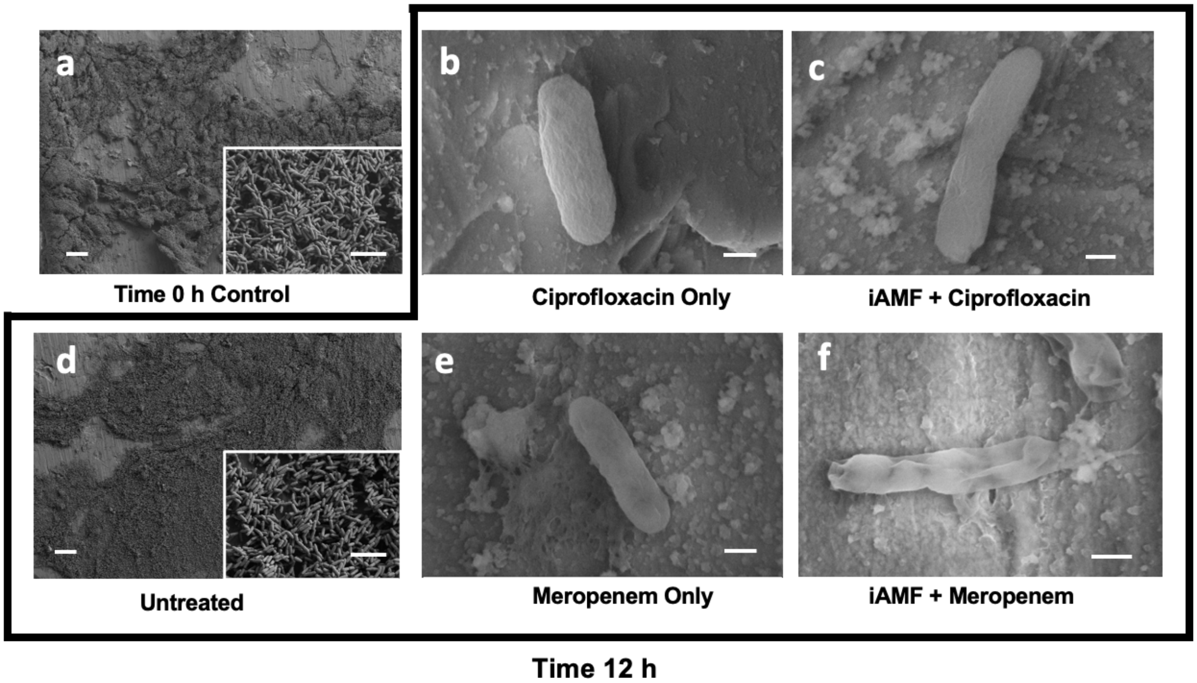

Supplementary Figure. 7 SEM images of antibiotic-resistant *P. aeruginosa* (MB699) biofilm treated with iAMF and antibiotics. Biofilm on a metal ring after treatment with iAMF (N_{exp}=12, T_{max}=65 °C, Δt_{exp} =5 min) and incubation in 64 µg mL^{-1} of meropenem or ciprofloxacin for 12 h. Magnification: 1250 × (**a** and **d**), 15000× (**a** and **d** inserts), 35,000× (**b, c, e,** and **f**). Scale bar: 300 nm (**b, c, e,** and **f**), 30 µm (**a** and **d**), 5 µm (**a** and **d** inserts)

Material	Density (kg m^{-3})	Electrical conductivity (S/m)	Relative Permittivity	Thermal Conductivity (W m^{-1} K^{-1})	Specific Heat (J $kg^{-1}\cdot K^{-1}$)	Perfusion (mL min^{-1} kg^{-1})	Metabolic Heat Generation (W kg^{-1})
316L stainless-steel	8000	1351351	1	16.3	500	-	-
Saline	1040	1.55	6750	0.570	3900	-	-
Muscle	1090	0.3840	6377.7	0.49	3421	37	0.91

Supplementary Table 1. Physical properties of materials used for simulation.

T_{max} (°C)	t_{exp} (s)	N_{exp}	Δt_{exp} (min)	Dosing Duration (h)	Dosing interval (h)
80	12*	1	5	--	12
65	3	12	5	1	12
50	1.2	24	5	2	12

Supplementary Table 2. iAMF parameters at different target temperature (T_{max}) . * 80 °C was achieved within 6 s of exposure and was held near this temperature for an additional 6 s with a proportional–integral–derivative (PID) calibration before stopping AMF.

Strain	Ciprofloxacin (μg mL^{-1})	Meropenem (μg mL^{-1})
**PAO1**	0.125	0.5
**MB699**	64	64

Supplementary Table 3. Minimum inhibitory concentrations of antibiotics used to treat strains of *P. aeruginosa.*
